# Does removal of federal subsidies discourage urban development? An evaluation of the US Coastal Barrier Resources Act

**DOI:** 10.1371/journal.pone.0233888

**Published:** 2020-06-30

**Authors:** Kyle Onda, Jordan Branham, Todd K. BenDor, Nikhil Kaza, David Salvesen

**Affiliations:** 1 Department of City and Regional Planning, University of North Carolina at Chapel Hill, Chapel Hill, North Carolina, United States of America; 2 Institute for the Environment, University of North Carolina at Chapel Hill, Chapel Hill, North Carolina, United States of America; University of Vermont, UNITED STATES

## Abstract

Urban development relies on many factors to remain viable, including infrastructure, services, and government provisions and subsidies. However, in situations involving federal or state level policy, development responds not just to one regulatory signal, but also to multiple signals from overlapping and competing jurisdictions. The 1982 U.S. Coastal Barrier Resources Act (CoBRA) offers an opportunity to study when and how development restrictions and economic disincentives protect natural resources by stopping or slowing urban development in management regimes with distributed authority and responsibility. CoBRA prohibits federal financial assistance for infrastructure, post-storm disaster relief, and flood insurance in designated sections (CoBRA units) of coastal barriers. How has CoBRA’s removal of these subsidies affected rates and types of urban development? Using building footprint and real estate data (*n =* 1,385,552 parcels), we compare density of built structures, land use types, residential house size, and land values within and outside of CoBRA units in eight Southeast and Gulf Coast states. We show that CoBRA is associated with reduced development rates in designated coastal barriers. We also demonstrate how local responses may counteract withdrawal of federal subsidies. As attention increases towards improving urban resilience in high hazard areas, this work contributes to understanding how limitations on infrastructure and insurance subsidies can affect outcomes where overlapping jurisdictions have competing goals.

## Introduction

Decades of US government policy have prompted extensive private development in hazardous coastal areas, where there is substantial risk to life and property [1]. In particular, federal financial assistance has been key to facilitating the construction of critical physical infrastructure, including highways and bridges, water supply and wastewater treatment facilities, beach stabilization projects, disaster assistance, and subsidized flood insurance. [2–4] After a major coastal storm or hurricane impacts a coastal barrier, federal disaster relief helps rebuild damaged properties and infrastructure. [5–7] Federal financial assistance has helped to perpetuate a cycle of coastal development, rising rates of hazard-related destruction, and subsidized post-disaster redevelopment. [4,8,9]

This study evaluates the long-term effects of withdrawing federal subsidies for urban infrastructure and flood insurance on urban development in sensitive coastal barriers. How effective are policies that aim to limit development in hazardous or environmentally sensitive areas by eliminating infrastructure and disaster recovery funding? How do these restrictions fare under management regimes with distributed authority and responsibility?

In this paper, we focus on the unique case created by the 1982 U.S. Coastal Barrier Resources Act (“CoBRA”; 16 U.S.C. 3501 et seq.) [10], which prohibits federal financial assistance (e.g., loans, grants, flood insurance, rebates, subsidies, or financial guarantees) for roads, bridges, utilities, erosion control, and post-storm disaster relief in statutorily designated sections of US coastal barriers. These areas, which we will call “CoBRA units,” comprise the John H. Chafee Coastal Barrier Resources System. [11]

Homeowners in CoBRA units are ineligible for subsidized flood insurance through the National Flood Insurance program (NFIP), while homeowners in adjacent, non-CoBRA areas are eligible. Moreover, CoBRA units may be subject to other types of development disincentives (e.g., additional subsidy restrictions) and land protections (e.g., zoning) enacted by other entities such as local and state government, private agencies, and other federal agencies. However, some CoBRA units may also be subject to development *incentives*, possibly the result of local governments replacing the federal subsidies removed by CoBRA.

The intent of this paper is to explore the impact of CoBRA on designated coastal barriers. In particular, we investigate the extent to which development has remained low in CoBRA units, in areas with other land use controls, and in areas with restrictions from both CoBRA and local land use controls. We also examine relationships between CoBRA and residential property values, and associations between development densities within and outside of CoBRA units.

We employ a cross-sectional approach to analyze differences in development across different combinations of development disincentives. We then compare distributions of building density, land use, house size, and land values across different combinations of development disincentives and regulations. Our study area extends 2 km inland from the coastlines of the eight Gulf Coast and Southeast states (Alabama, Florida, Georgia, Louisiana, Mississippi, North Carolina, South Carolina, and Texas). This area comprises 76% of all land in CoBRA units and 81% of land in Otherwise Protected Areas (OPA units), discussed below (Table 2).

Our analysis reveals a nuanced relationship between CoBRA and development patterns, including instances where the removal of federal subsidies may have been either counteracted or reinforced by state and local responses. This work has implications for understanding how the removal of development subsidies can affect desired outcomes in light of overlapping jurisdictions with competing goals and distributed authority and responsibility.

## Background

### Growth management and coastal development risk

There are many ways that government policy might be designed to reduce development risks, including attempts to restrict urban development in areas facing high risks of coastal hazards. Studies of urban management regimes and growth control policies have typically focused on understanding where development occurs and the characteristics of development in relation to urban services and targeted subsidy provisions. [12–14] However, much of this work has characterized growth management programs as being designed and implemented across large areas, often by a single agency, without considering heterogeneity in implementation.

In their classic study on implementation, Pressman and Wildavsky argue that programs fail because implementing agencies are thwarted by inter and intra organizational politicking and signaling after policies and programs have been adopted. [15] Within the context of large-scale infrastructure provision, multiple entities are often responsible for infrastructure financing and regulation, each of which may have competing agendas and different incentives. As a result, development patterns typically respond to multiple, regulatory and investment signals (e.g., US federal and state infrastructure funding) from overlapping and competing jurisdictions. [16–18] Few studies have explored instances where differential implementations of development management policies arise from interactions among jurisdictions at different levels (e.g., federal, state, and local). How effective are policies that aim to limit development in hazardous or environmentally sensitive areas by eliminating infrastructure and disaster recovery funding? How do these restrictions fare under management regimes with distributed authority and responsibility? Using the 1982 Coastal Barrier Resources Act as a case study, this study evaluates the long-term effects of withdrawing federally-funded urban infrastructure and flood insurance subsidies for development on sensitive coastal barriers.

### The 1982 U.S. Coastal Barrier Resources Act (“CoBRA”)

As an environmental policy, the 1982 U.S. Coastal Barrier Resources Act represents a novel vehicle for exploring the role of federal subsidies in promoting or inhibiting development in environmentally sensitive areas. CoBRA’s purpose is to 1) minimize loss of life, 2) reduce wasteful expenditures of federal revenues and 3) protect fish, wildlife, and other natural resources.

The prohibitions on federal expenditures went into effect immediately after the law’s passage (October 18, 1982), while those for federal flood insurance did not become effective until one year later (October 1, 1983). Congress initially designated 186 CoBRA units, totaling some 453,000 acres (~183323 ha) along 666 miles (~1072 km) of shoreline of the Atlantic and Gulf coasts. CoBRA was expanded and modified by Congress in 1990 to include “Otherwise Protected Areas” (OPAs), areas identified by Congress as being protected by other means (such as National and State parks), and for which federal subsidies other than flood insurance would be allowed. [19]

Flood insurance refers to the federally subsidized National Flood Insurance Program or “NFIP”. Communities that meet certain federal standards for floodplain management may participate in the NFIP. Homeowners and renters in participating communities are eligible to (voluntarily) purchase flood insurance from the Federal Emergency Management Agency (FEMA). Some 22,000 communities participate in the NFIP. [20] In addition, under FEMA’s Community Rating System, communities can implement activities that go beyond the minimum requirements of NFIP and in return, policyholders in those communities may qualify for discounts on their federal flood insurance premiums. As of 2017, over 1400 communities participate in CRS. [21]

Congress retains the sole authority to modify CoBRA unit boundaries upon the recommendation of the US Fish and Wildlife Service (FWS). Areas initially designated for inclusion were those (in 1982) with a) less than one walled and roofed building per five acres (~2 ha) of “fastland” (i.e., land above mean high tide), b) areas lacking urban infrastructure, vehicle access, water supply, wastewater disposal, and electric service to each lot, and c) areas that were not part of a development of 100 or more lots. In addition, designated units had to have at least one-quarter mile (0.4 km) of oceanfront. [22] Little community input was taken when designating units; some units were withdrawn from, and others added to, the system over time, with each change requiring an act of the US Congress. [23] Since the 1990 amendments, the Act has otherwise remained largely unchanged.

### CoBRA, policy resistance, and development pressure

Several studies have questioned the effectiveness of CoBRA. Investigations of random samples of CoBRA units by the United States Governmental Accountability Office (GAO) in 1992 and 2007 identified continuing development in many CoBRA units, which was facilitated by numerous, documented actions by local, state, and federal agencies. Case studies have discovered efforts by state and local governments to encourage development in CoBRA units, sometimes by substituting their own subsidies for those withdrawn by the federal government. [22,24–26] However, with the exception of the GAO’s 2007 study, no efforts have been made to comprehensively track or explain development in CoBRA units, and no studies have attempted to systematically account for other factors that may influence development in coastal areas, such as state or local development incentives or restrictions.

While the research available on CoBRA has been meager (particularly over the last ten years [27,28]), the act nevertheless provides the conceptual basis for considering analogues and generating hypotheses about the impact of CoBRA on development in designated coastal barriers. Retrospective analysis can now help understand how, for over 30 years, CoBRA has shaped development patterns.

While CoBRA does not regulate land use, it transfers some of the cost of development (e.g., infrastructure and flood insurance) to the private sector or to state and local governments. CoBRA designation is structurally similar to growth management instruments, such as urban service and growth boundaries, which have been widely used to restrict urban expansion and protect natural resources, such as farmland. [29,30] Urban service boundaries (USBs) do not prohibit development, but instead set expectations that services, such as sanitary sewers and water supply, are not publicly provided outside their specified areas. There is conflicting evidence regarding the effectiveness of USBs in containing low density urban expansion and requisite infrastructure development. [31,32]

Similarly, urban growth boundaries (UGBs) seek to restrict the area where development can occur in a jurisdiction. Like USBs, UGBs also preserve amenities (e.g. open space) whose value are internalized into higher land and housing prices, where development is allowed, [33] or increased development densities. [34–36] However, these effects are diluted when political pressure and built-in mechanisms for changes to UGB geographic delineations weaken the market signal intended to concentrate development intensity into core urban centers. [37,38] We contend that the same pressures in USBs and UGBs can occur in CoBRA units that border expanding urban areas—an effect that can mediated by regional development pressure (i.e., regional economic growth).

### Hypotheses

In this paper, we use a cross-sectional approach to analyze differences in development across different combinations of development disincentives. Since CoBRA units were almost exclusively designated in areas with development densities lower than one structure per five acres (~2 ha) as of 1982, there is the potential that land that was designated was unattractive for development in the first place and potentially correlated with becoming part of CoBRA. We do not attempt to resolve this endogeneity problem and instead present our findings as exploratory and descriptive.

**Fig 1:**
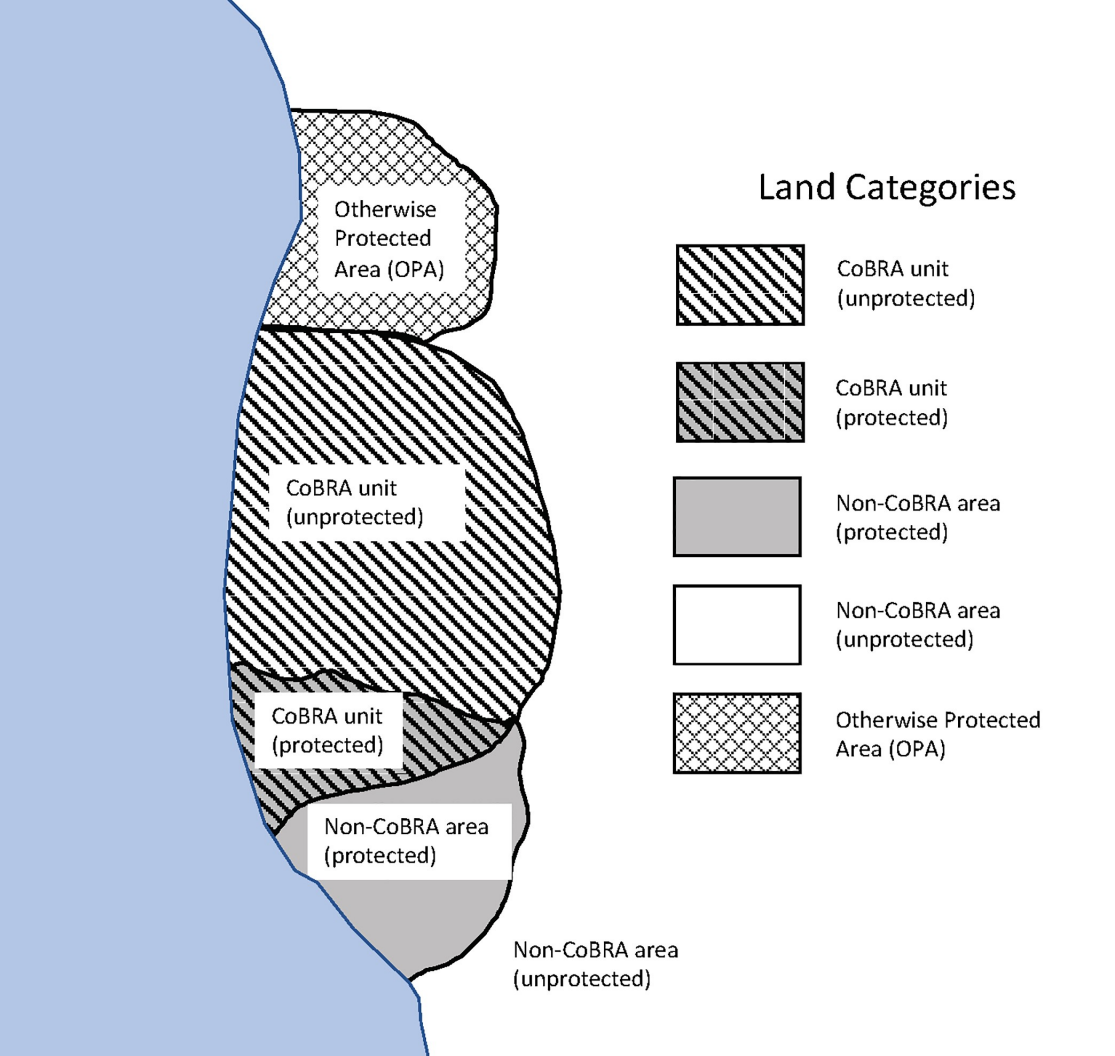
Depiction of land categories and their overlaps.

We conceptualize three levels of restrictions and regulations affecting coastal development (explained in more detail in the Study Area section), which can be configured to categorize coastal land into five categories or types, shown in Table 1 and depicted in Fig 1. The three restrictions include NFIP eligibility, other federal expenditures (e.g., for roads or sewer systems), and restrictions on urban development (e.g., designation as protected lands). Table 1 also shows our four hypotheses (H1 –H4) based on our five categories of land.

**Table 1:** Land categories by coastal development disincentive/regulations and hypotheses (H1-4).

Land Category	Eligible for flood insurance (NFIP)?	Eligible for other federal spending?	Is urban dev. un-restricted?	H1: CoBRA reduces dev. intensity	H2: CoBRA interacts with protected areas	H3: CoBRA creates a luxury effect for dev. parcels	H4: Dev. pressure spills into CoBRA units
Non-CoBRA area, unprotected (Type 1)	Yes	Yes	Yes				If high dev. rate, then Type 1&4 dev. rates are similar
Non-CoBRA area, protected (Type 2)	Yes	Yes	No	Less dev. than Type 1			
OPA (Type 3)	No	Yes	No	Less dev. than Type 1	More dev. than Type 5		
CoBRA unit, unprotected (Type 4)	No	No	Yes	Less dev. than Type 1		Dev. property values: higher than Type 1	
CoBRA unit, protected (Type 5)	No	No	No	Less dev. than Type 1	Less dev. than Type 2 and 4	Dev. property values: Higher than Type 2	

“Protected” status = areas specified in USGS Protected Areas Database, which includes lands protected or managed for purposes of government use, recreation, and habitat conservation. “dev.” = development or developed. OPA = Otherwise Protected Areas.

We aim to test four hypotheses (H1-4; Table 1). We first (H1) hypothesize that CoBRA affects land markets by increasing development costs (and therefore decreasing development extent) in CoBRA units as a result of higher, non-subsidized infrastructure and flood insurance costs. However, CoBRA’s impact might be weaker than outright protection through designation as a conservation area such as a park (e.g., by state or local government). Thus, we expect that parcels in unprotected CoBRA units and OPAs (as documented in the USGS Protected Areas Database, described below) experienced less extant overall development than non-CoBRA areas, where the CoBRA and non-CoBRA areas are not subject to other restrictions on land use.

Second (H2), we expect that withdrawal of federal subsidies acts synergistically with direct development restrictions (e.g., easements or other land use controls), resulting in less overall development in CoBRA units where the restrictions apply, than in CoBRA units where they do not. We also expect less development in protected CoBRA units than in protected non-CoBRA areas, as only the CoBRA units face the additional cost of non-federally subsidized infrastructure, disaster recovery, and flood insurance. The OPAs also offer a salient contrast, by providing explicit federal recognition of some, but not all areas with development restrictions, and withdrawing flood insurance subsidies from them. We expect land in OPAs, by virtue of being eligible for federal subsidies other than flood insurance, to be marginally more developed, than protected CoBRA units that are subject to restrictions, but not recognized as OPAs.

Third (H3), we expect there to be countervailing influences of CoBRA on property values. The withdrawal of federal subsidies under CoBRA, coupled with other development restrictions (e.g., easements or other land use controls), should tend to depress land values and increase development costs. However, in some cases, the low-density and secluded nature of land in CoBRA could make these areas attractive to development. [39] Under these circumstances, we suspect the property values for comparable properties may be higher in CoBRA units than in non-CoBRA areas.

Our final hypothesis (H4) concerns the regional heterogeneity and spatial dependence of CoBRA’s effects. In cases where development pressures are strong enough due to lack of developable land in neighboring, non-CoBRA areas, or where other actors–such as local or state governments–assume the burden of replacing foregone federal subsidies, we hypothesize that development rates in unprotected CoBRA units would resemble those in *proximate*, unprotected non-CoBRA areas. This suggests a range of potential situations, including CoBRA units that develop very little, if at all, and others that develop at comparable rates as nearby non-CoBRA areas.

## Materials and methods

### Study area

Our study concerns the coastline along the U.S Gulf Coast and Southeast Coast in eight states, Alabama, Florida, Georgia, Louisiana, Mississippi, North Carolina, South Carolina, and Texas (Table 2). In order to compare development patterns within CoBRA units to comparable non-CoBRA areas, we first restricted our study area to land within 2 km of our study states’ coastlines. Looking beyond areas proximate to coastal barriers could lead to statistical misspecification problems as significantly different economic and social dynamics affect inland and coastal barrier development patterns. [40]

**Table 2:** Extent of Coastal Barrier Resources System units (“CoBRA units”) and Otherwise Protected Areas (OPAs) in eight study states. Fastland refers to land above the mean high tide line.

	Unit count		Fastland (ha)		Shore length (km)	
	CoBRA	OPA	CoBRA	OPA	CoBRA	OPA
Alabama	4	6	3,586	6,333	33	27
Florida	68	63	54,354	116,809	375	375
Georgia	6	7	13,729	98,095	35	121
Louisiana	17	4	18,803	6,830	315	175
Mississippi	6	1	494	2,058	107	63
North Carolina	9	7	15,425	43,422	69	241
South Carolina	16	7	25,853	9,897	33	77
Texas	17	18	116,475	174,942	270	227
Sample total	143	113	248,719	458,387	1,242	1,306
Entire CBRS	585	277	329,215	566,040	2,282	2,042
% of entire system represented	24%	41%	76%	81%	54%	64%

We overlaid GIS shapefiles delineating CoBRA units and OPAs [41], as well as the USGS Protected Areas Database [42], which is a geospatial database of protected areas that are “…dedicated to the preservation of biological diversity and to other natural (including extraction), recreation and cultural uses, managed for these purposes through legal or other effective means.” This procedure resulted in land area being sampled and classified from 85 coastal counties, 77 of which contained at least one CoBRA unit.

Our primary units of analysis were individual land parcels. We retrieved these geospatial cadastral data from the National Parcel Data Portal, a proprietary aggregation of county-based georeferenced parcel polygons available from Boundary Solutions, Inc. [43] Nearly 46% of the total area of our study parcels is overlapped by CoBRA units or OPAs, and 62% of the total area is within a protected area or in a CoBRA unit. Using these overlays, we classified each parcel into one of the five development disincentive categories (Table 1): unprotected, non-CoBRA areas (Type 1), protected, non-CoBRA areas (Type 2), OPAs (Type 3), unprotected, CoBRA units (Type 4), and protected CoBRA units (Type 5). Where parcels were split by CoBRA units, OPAs, or protected areas, we classified the parcel as the category for which it had the greatest portion of its area within.

### Data

For parcels within our sample area, we sourced current property characteristics from the 2016 vintage of the ZTRAX transactions database produced by Zillow, which is made available to researchers upon request, subject to a Data Use Agreement. [44] Using county assessor parcel ID numbers, we matched ZTRAX real estate data to the parcel polygons. The variables retrieved from ZTRAX for this analysis include a nationally standardized land use code (which we summarize into eight different categories, as described in the “Land Use Comparison” sub-section), the year built of each structure on a parcel (if any), the most recent sales price in USD, the recording date of the most recent sale, and the square footage of each structure on the parcel (see S1 Table).

In order to create an inventory of development density, we counted structures within parcels and measured the percentage of each parcel covered by structures using spatial intersection queries from the *sf* package in the R statistics software [45]. Our source for structures and building footprints is a dataset of 125,192,184 computer generated building footprints in all 50 US states in GeoJSON format, which was produced and distributed by Microsoft, Inc. under the Open Data Commons Open Database License [46]. These footprints were generated from high-resolution aerial photographs taken between 2014 and 2016.

### Land Use Comparison

Using ZTRAX’s nationally harmonized land use categorization, we classified each parcel into one of eight summary land use categories: *government/military*, *residential-single family*, *residential-multifamily*, *other developed*, *open space*, *agriculture*, *zoned vacant lots*, and *other or not classified*. We then computed the proportional cross-tabulation of total area represented by parcels by development disincentive category (see Table 1) and by land use category.

*Government/military* includes government offices, military facilities, and government-owned land restricted to the public. Urban or developed land uses are divided into *Residential-single family*, *residential-multifamily*, *and other-developed land uses*. *Residential-single family* includes detached residences and mobile homes. *Residential-multifamily* includes apartments, duplexes, townhomes, condominiums, and mobile homes. *Other-developed* includes all other kinds of development other than government or agricultural, including non-governmental institutions, commercial, industrial, and recreational structures.

Undeveloped land has many land use categories represented in ZTRAX, which we aggregate to *open space*, *agriculture*, *zoned vacant lots*, and *other or not classified* land uses. *Open space* refers to land designated as parks, conservation areas, and similar open space areas with defined land uses. *Agriculture* refers to any agricultural use. *Zoned vacant lots* refer to parcels that have been zoned for–and are often surrounded by–residential, commercial, industrial, or institutional structures, but have no structures on them. *Other or not classified* parcels do not have designated land uses and generally represent unused, undeveloped, but not necessarily protected land.

### Parcel characteristic comparison

We estimated a series of linear regression equations of the form in Eq 1.

$$y_{ij}=\alpha_{j}+\Sigma \beta C_{i}+\epsilon$$ (1)

In Eq 1, *i* indicates parcel, *j* indicates county, *α_j_* indicates county fixed effects, and ***C*** is a vector of dummy variables indicating whether parcel *i* is in each of the development disincentive categories. These regressions constructed confidence intervals around the difference in the average value of *y* between parcels in unprotected, non-CoBRA areas (Type 1; base category) and each of the other categories. We estimated regression equations for five dependent variables:

1. % area of parcel covered by structures [for only developed parcels (parcels with at least one structure)]. This is a relative measure of building form and extent.
2. % area of parcel covered by structures [for all parcels]. This is the measure we use to determine and generalize development extent and/or development rate.
3. log(residential area (m^2^)) [residential units only]. This measure indicates relative housing size.
4. log(most recent sales price (inflation adjusted to 2016 USD)/(residential area (m^2^))) [residential units only]. This measure normalizes land values to residential units per area.
5. (residential area (m^2^))/(parcel area (m^2^)) [residential units only]. This measure normalizes residential construction extent at the parcel level.

We estimated these regressions with and without county fixed effects to assess if different patterns emerge at an overall level or when controlling for local conditions. We also estimated a series of Hierarchical Linear Models (HLM) as a check on the robustness on our models with county fixed effects; we fit a multi-level random intercept model, with parcels nested in counties and counties nested in states.

$$y_{ics}=\alpha_{cs}+\Sigma \beta_{i}T_{i}+\epsilon_{ics}$$

$$\alpha_{cs}=\gamma_{s}+u_{cs}$$

$$\gamma_{s}=\delta +\eta_{s}$$

Where T_i_ are the treatments of interest and c and s refer to counties and states respectively. We find no substantial differences in estimates or significance levels and therefore choose to present our fixed effects results given their ease of interpretation. The results of the HLM are presented in S3 Table.

### Regional heterogeneity

We probed for regional heterogeneity in the patterns of development within and outside of CoBRA units by conducting a cluster analysis at the county level (on land within each county that is within the study area, excising land that is outside the study area). Our aim was to explore whether there was variation in the development of land in CoBRA units compared with neighboring, non-CoBRA areas.

We first removed from counties any area that is open water, although we left wetlands, which have been dredged and filled for development in many areas. To remove open water from county polygons, we employed the 2016 National Land Class Dataset (NLCD), whose 30m raster land cover data is consistent across the United States. [47] For each county that has at least one CoBRA unit, we construct the following variables:

1. The inverse hyperbolic sine transform (arcsinh) of (structures/hectare in non-CoBRA areas)
2. The inverse hyperbolic sine transform (arcsinh) of structures/hectare in CoBRA units and strutures/hectare in OPAs
3. Proportion of area in CoBRA that is designated as OPA (for the rest of our analysis, we consider OPA and CoBRA units to be exclusive)

We then standardized these variables to a common scale, constructed a distance matrix based on Manhattan distances, and clustered the counties with Ward’s hierarchical clustering algorithm. [48] We used the resulting dendrogram to generate three clusters corresponding to distinct development patterns across system and non-CoBRA areas within counties.

Data management and analysis was performed in ArcGIS 10.4 and the R statistical software [49], with geospatial data processing in R performed using functions from the *sf* package [45]. Maps were produced in ArcGIS 10.4, and figures produced with R using ggplot2. Replication code and data (excepting restricted parcel boundaries and data) are available from the UNC Dataverse. [50]

## Results and discussion

Overall, we found that development rates remained lower and qualitatively different in CoBRA units compared to non-CoBRA areas (H1). However, there are significant outliers (H3 and H4). As of 2016, 34 of the 257 CoBRA units (13%) in our study area had development densities that would have precluded their designation if CoBRA were enacted today (i.e., greater than one dwelling unit per five acres [~2 ha] of fastland).

This pattern of local exceptions to a generally effective policy is consistent with heterogeneity in program implementation or effectiveness (H4). Effectiveness could be undermined by federal agencies circumventing or otherwise ignoring CoBRA, state and local government agencies filling in subsidy gaps (H3), or by developers providing infrastructure directly. [20–23] Alternatively, a relatively ineffective policy may be strengthened by complementary actions of other agencies.

**Fig 2:**
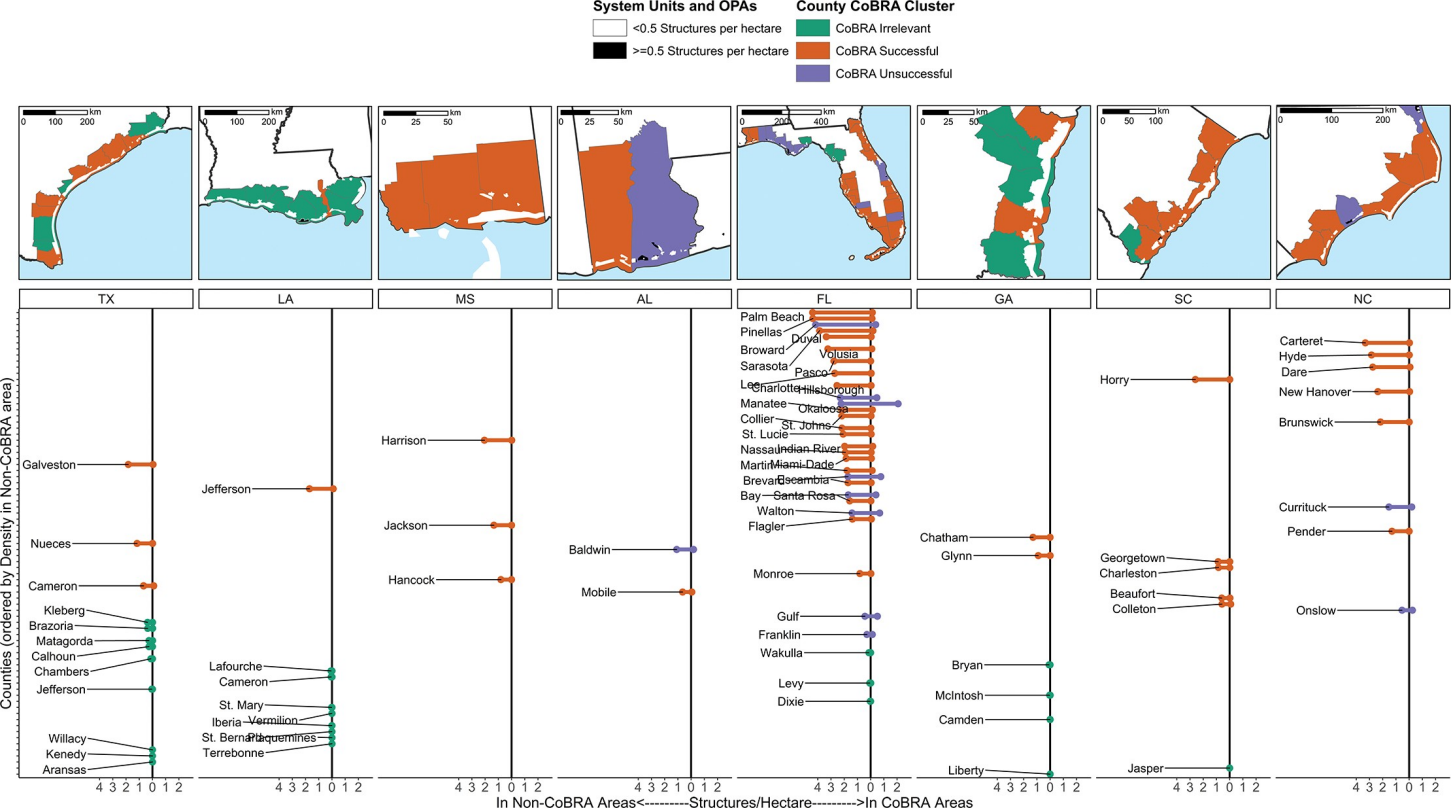
**Density of built structures (2016) in CoBRA units (right) and non-CoBRA areas (left) by county in each of in each of the eight study states.** Although our analysis extent covers only areas within 2 km of the coastline in counties with CoBRA units, for legibility this figure depicts the entire counties that were part of our analysis. CoBRA units in white or black (for high density) Legend coloring in both panels depicts results of cluster analysis of development rates (*n = 77* counties with CoBRA units) into three categories, where CoBRA could be identified as Successful, Unsuccessful, and Irrelevant.

Cluster analysis of the 77 counties in our eight states with CoBRA units identified three types of counties describing general patterns of development densities within and outside of CoBRA units, suggesting substantial regional variability in the effectiveness of CoBRA (H3 and H4; Fig 2).

Twenty-one counties formed a cluster that we call, “CoBRA Irrelevant,” where coastal development density in both CoBRA units and adjacent non-CoBRA areas was very low, suggesting an absence of development pressure that could have yielded significant development. Another 45 counties formed a cluster we call, “CoBRA Successful,” characterized by near-zero development in CoBRA units and significant development in nearby non-CoBRA areas (H1). We use the term “successful” here cautiously, as the CoBRA units in these areas may represent land that was particularly costly or unsuitable for development with or without the federal funding prohibited by CoBRA. However, despite this endogeneity concern, this set of counties can be characterized as having a strong difference in development rates within and outside of CoBRA units between 1980 and 2016.

The remaining 11 counties formed a cluster characterized by significant development within system-units relative to nearby non-CoBRA areas, which we refer to as, “CoBRA Unsuccessful.” Notably, these 11 counties exist entirely in Florida (8), Alabama (1), and North Carolina (2) (see maps in Fig 2). While this clustering within three states may indicate overriding roles played by specific state policies, it is important to note that, in these states, there are an additional 30 counties with CoBRA units that remain undeveloped. Additionally, it is difficult to study state-level impacts without having high-quality data on policy changes over time (as state policies affecting activities in CoBRA units have been dynamic), which is beyond the scope of the current study.

The counties in this “CoBRA Unsuccessful” cluster exhibited a wide range of development densities in non-CoBRA areas, from among the densest (e.g., 10.28/ha in Broward County, FL) to the sparsest (e.g., 1.07/ha in Gulf County, FL). It is possible that, in counties with lower densities in non-CoBRA areas, CoBRA units held more developable or desirable land than the non-CoBRA areas, such that the incentives to develop could override the lack of federal subsidies (H3/H4). However, since a similar dynamic does not regularly appear in the highest-density counties, most of which are in the “CoBRA successful” cluster (see Fig 2B), development pressure spillovers from proximate land may not be the primary driver of the development of CoBRA units (H4). Instead, we hypothesize that a combination of local conditions, including the actions of state and local government agencies, may play significant roles (H4).

One important policy that appears to interact with CoBRA-related federal funding withdrawals is federal, state and local direct development restrictions (H2). Within non-CoBRA areas, the average parcel size is six-times larger in protected areas than unprotected areas, suggesting that protection could be discouraging the subdivision of land that generally precedes urban development (Table 3).

**Table 3:** Areal extent (within study zone extending 2 km inland from coastline) and sample size of parcels in five development disincentive categories (from Table 1).

Category	Area (ha)	Coverage of study area (%)	Parcels (count)	Average parcel size (ha)
Non-CoBRA area, unprotected (Type 1)	459,905	38	1,228,760	0.3
Non-CoBRA area, protected (Type 2)	195,473	16	110,886	1.8
OPA (Type 3)	244,823	20	9,196	26.6
CoBRA unit, unprotected (Type 4)	243,994	20	21,879	11.2
CoBRA unit, protected (Type 5)	76,769	6	14,831	5.2
Total	1,220,964	100	1,385,552	0.9

An alternative explanation is that development restrictions that we characterize in this study as “protection,” create a luxury effect (H3) that tends to incentivize development of homes on larger lots. [36] CoBRA may act in complementary ways, as average parcel sizes in CoBRA units and OPAs are 2.5–15 times larger than parcels in protected, non-CoBRA areas. OPAs, which combine federal flood insurance program prohibitions with notable federal and state protections such as State and National Parks, Wildlife Refuges, and military installations, have the largest average parcel sizes. However, this may be almost completely endogenous, as such large protected parcels that are unlikely to be sold to private developers were the most likely to be designated as OPAs in the first place.

**Fig 3:**
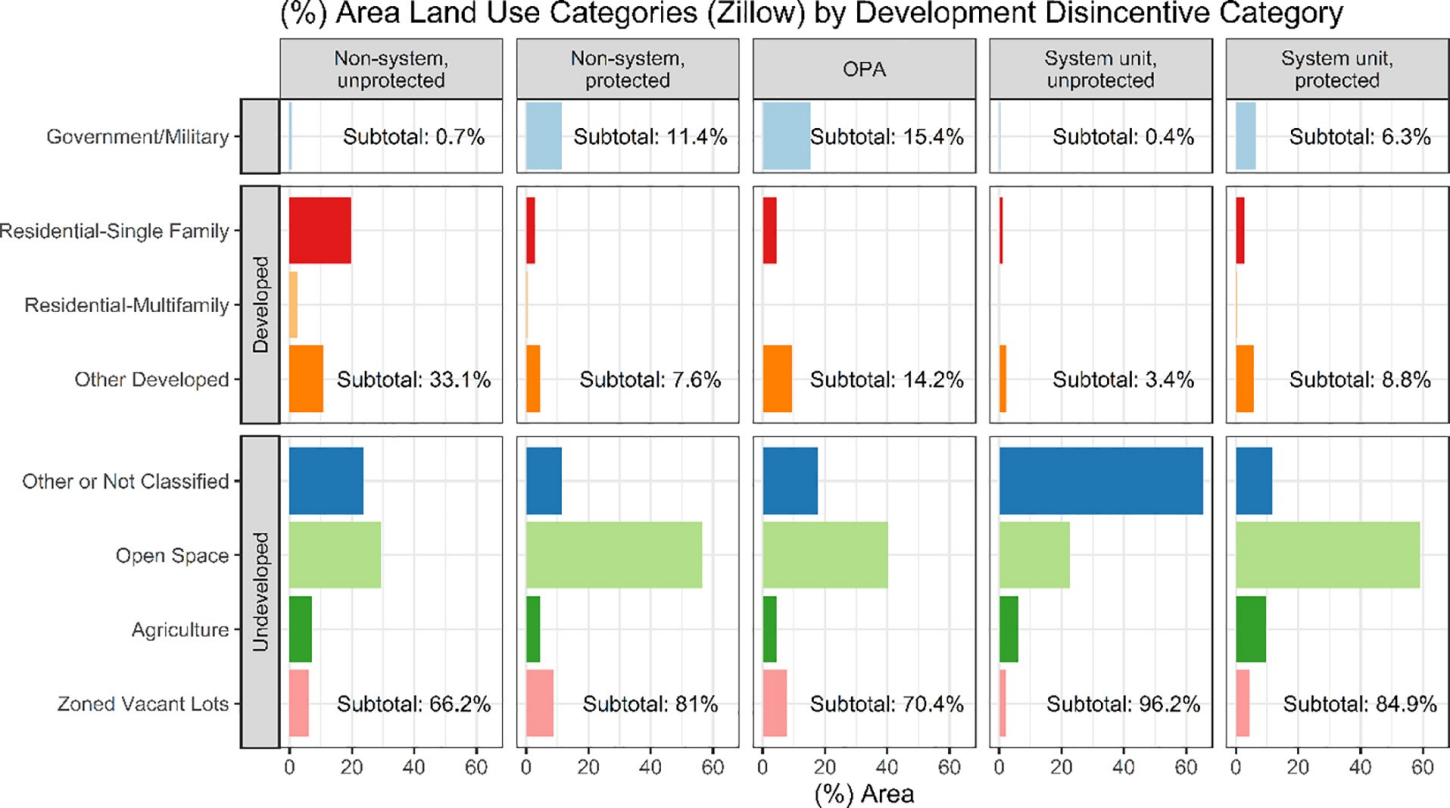
Relative extents of different types of land cover and land use among development disincentive categories. Urban land use is aggregated into *single-family residential*, *multifamily residential*, and *other developed* (including unitary parcels of mobile home parks, planned unit developments, and institutional residences). Undeveloped land use is aggregated into *open space* (designated parks, wildlife areas, conservation areas etc.), *agriculture* (any agricultural use), *zoned vacant lots* (referring to vacant lots that are nevertheless zoned to permit residential, commercial, industrial, or institutional land uses), and *other or not classified* (where parcels do not have designated land uses, they are generally not formally parcelized by county tax assessors and represent undeveloped and unused land). Government- and military-owned land may or may not have structures, but are generally exempt from local government development restrictions as well as some CoBRA subsidy restrictions.

Parcels in protected CoBRA units are almost three-times larger, on average, than parcels in protected, non-CoBRA areas. This finding is consistent with federal subsidy withdrawal increasing development costs (H1) and thereby discouraging subdivision and development to a greater degree than local protections, such as park designations, do on their own (H2). When accounting for the land uses on these parcels (Fig 3), the interaction between CoBRA regulations and protection becomes clearer. When comparing protected, non-CoBRA areas with protected CoBRA units, most land in unprotected units is “Not Classified” or “Other,” indicating unparcelized and undeveloped land owned by states, counties, and municipalities, but not designated for any particular land use. In contrast, land in protected units is mostly designated as open space, such as parks. This could have important ramifications for future development patterns. It is possible that local protections (as opposed to large-scale state and federal protections, typically represented in the OPA category) have been prompted by CoBRA designation itself (H2), particularly in areas that were otherwise attractive for development. This is an avenue for future study leveraging historical land ownership and protection records.

**Fig 4:**
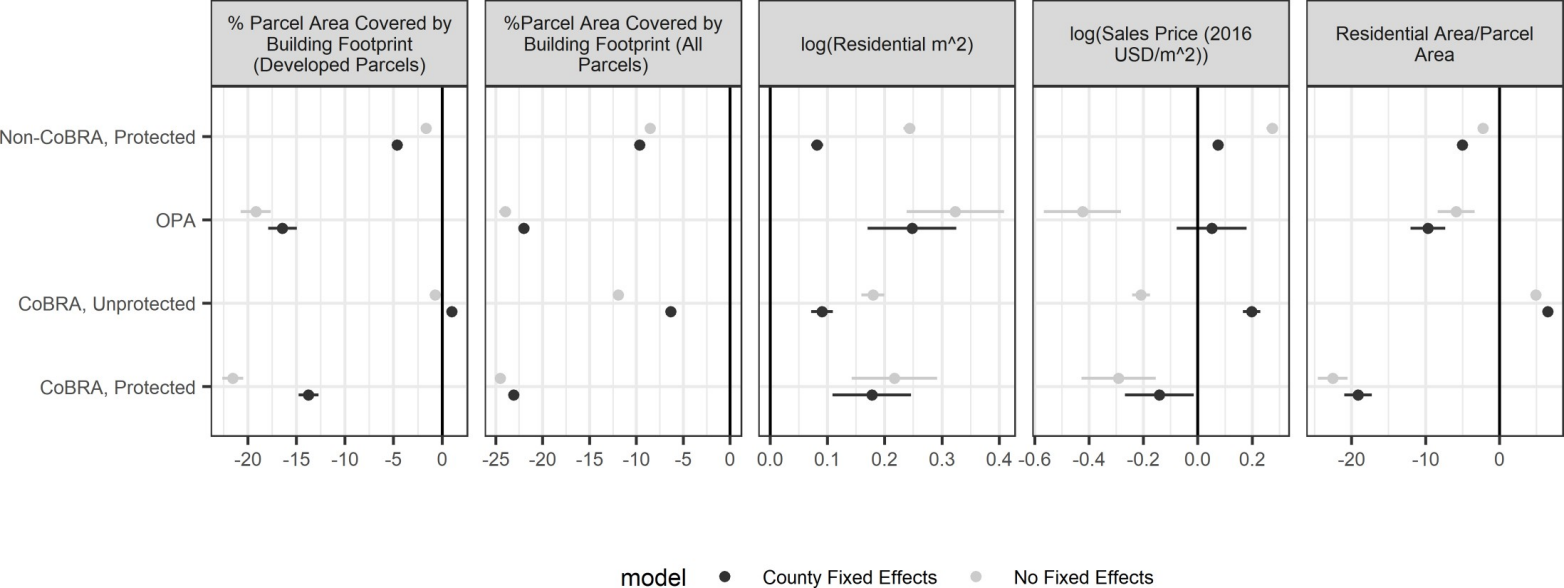
Regression results. Dependent variables: (a) The percentage of parcel covered by structure footprints, among only parcels with structures. (b) The percentage of parcel covered by structure footprints, among all parcels. (c) The natural logarithm of residential area (sq. m.) among parcels with residential land uses. (d) The natural logarithm of the most recent inflation-adjusted sales price per square meter for residential parcels. (e) The residential living area divided by the parcel area for residential parcels. Parcels are units of analysis. The base category is *non-CoBRA*, *unprotected* areas (Type 1). Dots represent point estimates and error bars indicate 95% confidence intervals. The x-axis is the effect size (i.e., the average difference between parcels in the indicated category from parcels in the base category). County fixed effects refer to regressions that use dummy variables to control for the county in which parcels are located.

We present regression results (Fig 4; for full regression results see S2 Table) with and without county fixed effects. The regressions without fixed effects show estimated average differences between each development disincentive category and unprotected, non-CoBRA areas across the entire sample. The regressions with county effects show the average of estimated differences between the categories *within each county*, thus accounting for differences between counties in the overall levels of each of the outcome metrics.

Among “developed” parcels (i.e., containing structures), unprotected CoBRA units (Type 4) show statistically ambiguous differences with unprotected, non-CoBRA areas (Type 1) in terms of percentage of land area covered by structures (H1; Fig 4A). Overall, unprotected CoBRA units experience slightly reduced development intensity (1% less parcel area covered by structures). However, when controlling for the county of observations (county fixed effects) CoBRA units experience slightly higher (1% more parcel area covered by structures) development intensity than non-CoBRA areas. This indicates that on average, parcels in unprotected CoBRA units with any development tend to have structures with smaller footprints relative to the parcel size than non-CoBRA areas, but this may have to do with correlations between the type of development that occurs in CoBRA units and overall development pressure and density in counties. When controlling for county (i.e., when comparing more spatially proximate CoBRA units and non-CoBRA areas), the relationship is reversed, indicating that in a given region, parcels in CoBRA units tend to have a larger proportion of their area covered by structures.

Protection is associated with reduced development intensity on built-upon parcels in non-CoBRA areas by less than 5 percent, while protection in CoBRA units is associated with 12–25 percent increases in intensity (H2; Fig 4B). This interaction between CoBRA and other protections is even stronger when considering residential area densities (Fig 4E), where protected CoBRA units (Type 5) have lower densities than any other category, while unprotected CoBRA units (Type 4) have the highest residential densities. Thus, in this respect, protection appears to strengthen the impact of CoBRA and is associated with a reduction in development intensity when it does occur (H2).

This finding highlights a way in which federal policies, such as CoBRA, can be strengthened by the regulatory actions of other agencies. While CoBRA and other protections are independently associated with reductions in development and development intensity, when parcels have both types of policies applied, they experience even lower development intensity on average. That is, localities wishing to limit development in coastal barriers may find more success applying policy tools such as zoning and limits on infrastructure in CoBRA areas than non-CoBRA areas.

However, CoBRA units and all protected areas experience larger average house sizes (log[m^2^]; Fig 4C). Moreover, controlling for individual counties (fixed effects), CoBRA unit designation is associated with significantly higher property sales prices (Fig 4D). Thus, while residential development in CoBRA units is less common than in non-CoBRA areas, the development that does occur tends to be of larger, more expensive houses, which suggests luxury effects (H3). This is consistent with the literature on the impact of parks and natural areas on property values, wherein such amenities are valued by consumers, and this value is expressed in sales prices [51]. We speculate that CoBRA might affect property values and development by providing large natural amenities with relatively low development intensity. It is also possible that, by restricting NFIP eligibility, any development that occurred in CoBRA units was necessarily initiated by those who could afford alternative insurance coverage, and that the income or wealth required to do so is correlated with willingness and ability to pay for larger and/or more expensive properties.

When controlling for protected status and system designation across all parcels in our study area, unprotected, non-CoBRA areas (Type 1) experience higher development intensities (% parcel covered by built structures) than unprotected CoBRA units (Type 4; H1; Fig 4B). Protected parcels in non-CoBRA areas experience a similar decrease in development intensity as unprotected parcels in CoBRA units. OPAs and protected CoBRA units are developed much less intensively than all other categories (H2), although much of this effect is likely due to the endogenous designation of CoBRA units in previously undeveloped areas.

## Conclusion

Our results suggest strong relationships between CoBRA designation and resulting development density and land use. However, these results should not be interpreted causally due to the endogeneity with which CoBRA units were drawn around undeveloped areas. Even so, CoBRA designation is associated with lower development density, higher proportions of vacant land, and larger average house size relative to non-CoBRA areas. This confirms much of our first hypothesis, H1: CoBRA is associated with lower development rates.

Moreover, the differences in house size, development propensity, and house sales prices (when controlling for the county) within CoBRA relative to unprotected areas outside CoBRA appear to be indistinguishable from the differences observed as a result of independent development restrictions initiated by other federal agencies and non-federal actors in areas such as parks, wildlife refuges, or conservation areas. This confirms parts of our second hypothesis (H2: CoBRA interacts with protected areas), and even suggests that CoBRA designation appears to have similar outcomes as designation as a protected area. Moreover, we provide evidence that CoBRA and other protections applied together may reduce development more than either alone.

While CoBRA designation shifts infrastructure costs to the private sector, our finding that protection and CoBRA are associated with equally expensive homes, suggests that CoBRA may create a strong seclusion effect that incentivizes luxury development patterns representing substantial property risk in coastal barriers. [39] However, our county fixed effects models demonstrate that this effect–which we suggested in our third hypothesis (H3: CoBRA creates a luxury effect for developed parcels)–may be mitigated at the community level. This regionally-dependent behavior suggests that the luxury effect may be mitigated by direct development restrictions, highlighting the potential importance of state and local land use policy in enabling, complementing, or counteracting federal policy goals.

Within the same county, there does not appear to be a direct relationship between the extent of development in CoBRA units and non-CoBRA areas. Counties with high development in CoBRA units do not necessarily have high non-CoBRA development rates, and many highly developed counties have little to no development in CoBRA units. This suggests that the primary determinant of development in CoBRA units is not scarcity of developable land in non-CoBRA areas. One possible explanation is that more complex spatial and political relationships are at play, rather than simply the spillover effects of our fourth hypothesis (H4; That is, in areas with high development pressure, development will eventually spill into CoBRA units).

In lieu of a direct spillover effect, we speculate that high development rates in CoBRA units could instead be the result of local or state development policies or subsidy substitutions. To determine this exact relationship, future work should consider the timing and spatial dependencies of development and policy within and around CoBRA units. This same work should consider the roles of changing state-level policies as well.

Is CoBRA achieving its statutory objective of reducing development in designated coastal barrier areas? Our results suggest that CoBRA has been successful in decreasing development rates and the total amount of development–the vast majority of CoBRA units remain undeveloped. Likewise, independent protection of coastal barriers has also been effective. However, CoBRA designation and other forms of protection appear to interact in preventing development, decreasing land values, and development densities. The particular regulatory mechanisms that may be complementary of, or offsetting to, CoBRA need to be investigated more fully with studies tracing local policies and development over time.

## Supporting information

S1 Table Parcel characteristics retrieved from ZTRAX. (DOCX)

S2 Table Regression results. Each dependent variable has two regression models, one without (odd numbered) and one with (even numbered) county fixed effects. Coefficients represent mean differences in the dependent variable (columns) between development disincentive category (rows) and base category of non-CoBRA, unprotected land (Type 1). Standard errors shown below coefficients in parentheses. * p<0.1, ** p<0.05, ***p<0.01.
(DOCX)

S3 Table Hierarchical linear regression results. Coefficients represent mean differences in the dependent variable (columns) between development disincentive category (rows) and base category of non-CoBRA, unprotected land (Type 1). Standard errors shown below coefficients in parentheses. * p<0.1, ** p<0.05, ***p<0.01.
(DOCX)
