# Supplementary material for: Does removal of federal subsidies discourage urban development? An evaluation of the US Coastal Barrier Resources Act
Source: PLoS One. 2020 Jun 30;15(6):e0233888. doi: 10.1371/journal.pone.0233888 (PMC7326218; doi:10.1371/journal.pone.0233888)
Supplement: S1 Table — (DOCX) [file pone.0233888.s001.docx]

Supplementary Table 1: Parcel characteristics retrieved from ZTRAX

| Variable | Notes |
| --- | --- |
| AssessorParcelNumber | ID key used to match ZTRAX data to parcel polygons from NPDP |
| PropertyLandUseStndCode | An alphanumeric land use code corresponding to a more detailed land use description |
| YearBuilt | The year the structure (for which multiple may exist for one parcel) was constructed |
| SalesPriceAmount | The most recent sales price |
| RecordingDate | The date the most recent sale was recorded at the applicable county office. Sales may be recorded months after the actual transfer takes place, but the field for the actual sale date was completely missing for parcels in our study area. |
| BuildingAreaSqFt | The square footage of the structure as considered for real estate transactions. Generally “finished” square footage. |
