# Supplementary material for: Does removal of federal subsidies discourage urban development? An evaluation of the US Coastal Barrier Resources Act
Source: PLoS One. 2020 Jun 30;15(6):e0233888. doi: 10.1371/journal.pone.0233888 (PMC7326218; doi:10.1371/journal.pone.0233888)
Supplement: S2 Table — Each dependent variable has two regression models, one without (odd numbered) and one with (even numbered) county fixed effects. Coefficients represent mean differences in the dependent variable (columns) between development disincentive category (rows) and base category of non-CoBRA, unprotected land (Type 1). Standard errors shown below coefficients in parentheses. * p<0.1, ** p<0.05, ***p<0.01. (DOCX) [file pone.0233888.s002.docx]

Supplementary Table 2: Regression results. Each dependent variable has two regression models, one without (odd numbered) and one with (even numbered) county fixed effects. Coefficients represent mean differences in the dependent variable (columns) between development disincentive category (rows) and base category of non-CoBRA, unprotected land (Type 1). Standard errors shown below coefficients in parentheses. * p<0.1, ** p<0.05, ***p<0.01

|  | Structure footprint/parcel area (%) [parcels with buildings only] | | Structure footprint/parcel area (%) [all parcels] | | log(Residential area) (m^2^) | | Residential area / parcel area (%) | | log(Sales price (2016 USD)/residential area (m^2^) | |
| --- | --- | --- | --- | --- | --- | --- | --- | --- | --- | --- |
|  | (1) | (2) | (3) | (4) | (5) | (6) | (7) | (8) | (9) | (11) |
| County fixed effects? | No | Yes | No | Yes | No | Yes | No | Yes | No | Yes |
| Non-CoBRA, protected (Type 2) | -1.666^***^ | -4.663^***^ | -8.542^***^ | -9.672^***^ | 0.243^***^ | 0.082^***^ | -2.253^***^ | -5.042^***^ | 0.274^***^ | 0.074^***^ |
|  | (0.11) | (0.11) | (0.09) | (0.09) | (0.01) | (0.01) | (0.13) | (0.13) | (0.01) | (0.01) |
| OPA (Type 3) | -19.184^***^ | -16.442^***^ | -23.997^***^ | -21.997^***^ | 0.323^***^ | 0.248^***^ | -5.861^***^ | -9.692^***^ | -0.425^***^ | 0.051 |
|  | (0.79) | (0.75) | (0.33) | (0.31) | (0.04) | (0.04) | (1.27) | (1.19) | (0.07) | (0.07) |
| CoBRA unit, unprotected (Type 4) | -0.729^***^ | 0.963^***^ | -11.944^***^ | -6.352^***^ | 0.180^***^ | 0.091^***^ | 4.924^***^ | 6.538^***^ | -0.209^***^ | 0.198^***^ |
|  | (0.26) | (0.25) | (0.19) | (0.18) | (0.01) | (0.01) | (0.29) | (0.28) | (0.02) | (0.02) |
| CoBRA unit, protected (Type 5) | -21.551^***^ | -13.754^***^ | -24.518^***^ | -23.082^***^ | 0.217^***^ | 0.177^***^ | -22.563^***^ | -19.139^***^ | -0.292^***^ | -0.141^**^ |
|  | (0.55) | (0.53) | (0.22) | (0.21) | (0.04) | (0.04) | (1.02) | (0.94) | (0.07) | (0.06) |
| Intercept | 31.581^***^ | 18.419^***^ | 25.9^***^ | 14.234^***^ | 7.548^***^ | 7.422^***^ | 33.103^***^ | 18.884^***^ | 4.937^***^ | 5.044^***^ |
|  | (0.02) | (0.11) | (0.02) | (0.10) | (0.00) | (0.00) | (0.02) | (0.12) | (0.00) | (0.01) |
| Observations | 1,121,063 | 1,121,063 | 1,406,187 | 1,406,187 | 587,586 | 587,586 | 909,381 | 909,381 | 352,385 | 352,385 |
| Adjusted R^2^ | 0.002 | 0.158 | 0.021 | 0.206 | 0.004 | 0.185 | 0.001 | 0.179 | 0.002 | 0.188 |
| F Statistic | 591^***^ | 2,799^***^ | 7,534^***^ | 4,606^***^ | 617^***^ | 2,783^***^ | 278^***^ | 2,825^***^ | 217^***^ | 2,210^***^ |
