# Supplementary material for: Does removal of federal subsidies discourage urban development? An evaluation of the US Coastal Barrier Resources Act
Source: PLoS One. 2020 Jun 30;15(6):e0233888. doi: 10.1371/journal.pone.0233888 (PMC7326218; doi:10.1371/journal.pone.0233888)
Supplement: S3 Table — Coefficients represent mean differences in the dependent variable (columns) between development disincentive category (rows) and base category of non-CoBRA, unprotected land (Type 1). Standard errors shown below coefficients in parentheses. * p<0.1, ** p<0.05, ***p<0.01. (DOCX) [file pone.0233888.s003.docx]

Supplementary Table 3: Hierarchical Linear Regression results. Coefficients represent mean differences in the dependent variable (columns) between development disincentive category (rows) and base category of non-CoBRA, unprotected land (Type 1). Standard errors shown below coefficients in parentheses. * p<0.1, ** p<0.05, ***p<0.01

|  | *Dependent variable:* | | | | |
| --- | --- | --- | --- | --- | --- |
|  |  | | | | |
|  | Structure footprint/parcel area (%) [parcels with buildings only] | Structure footprint/parcel area (%) [all parcels] | log(Residential area) (m^2^) | Residential area / parcel area (%) | log(Sales price (2016 USD)/residential area (m^2^) |
|  | (1) | (2) | (3) | (4) | (5) |
|  | | | | | |
| Non-CoBRA, protected (Type 2) | -4.653*** | -9.667*** | 0.082*** | -5.034*** | 0.074*** |
|  | (0.110) | (0.089) | (0.005) | (0.126) | (0.010) |
|  |  |  |  |  |  |
| OPA (Type 3) | -16.494*** | -22.004*** | 0.248*** | -9.716*** | 0.051 |
|  | (0.749) | (0.304) | (0.039) | (1.192) | (0.065) |
|  |  |  |  |  |  |
| CoBRA unit, unprotected (Type 4) | 0.948*** | -6.362*** | 0.091*** | 6.537*** | 0.198*** |
|  | (0.252) | (0.179) | (0.010) | (0.278) | (0.016) |
|  |  |  |  |  |  |
| CoBRA unit, protected (Type 5) | -13.756*** | -23.072*** | 0.177*** | -19.124*** | -0.143** |
|  | (0.526) | (0.213) | (0.035) | (0.943) | (0.064) |
|  |  |  |  |  |  |
| Constant | 21.045*** | 15.146*** | 5.135*** | 22.780*** | 7.184*** |
|  | (3.625) | (3.086) | (0.117) | (3.502) | (0.101) |
|  |  |  |  |  |  |
|  | | | | | |
| Observations | 1,121,063 | 1,406,187 | 587,586 | 909,381 | 352,385 |
|  | | | | | |
